# Physical literacy in older adults: a scoping review protocol

**DOI:** 10.3389/fpubh.2023.1217773

**Published:** 2024-01-18

**Authors:** Carmen Galán-Arroyo, Javier de los Ríos-Calonge, Jorge Rojo-Ramos, Jose A. Parraca, Cesar Fonseca, Antonio Castillo-Paredes, Marco Alexandre da Silva Batista

**Affiliations:** ^1^Physical and Health Literacy and Health-Related Quality of Life (PHYQoL), Faculty of Sport Science, University of Extremadura, Cáceres, Spain; ^2^Sport, Health and Exercise Research Unit (SHERU), Department Sport and Well-Being, Castelo Branco Polytechnic Institute, School of Education, Castelo Branco, Portugal; ^3^Department of Sport Sciences, Sports Research Centre, Miguel Hernández University of Elche, Elche, Spain; ^4^Physical Activity for Education, Performance and Health, Faculty of Sport Sciences, University of Extremadura, Cáceres, Spain; ^5^Departamento de Desporto e Saúde, Escola de Saúde e Desenvolvimento Humano, Universidade de Évora, Évora, Portugal; ^6^Comprehensive Health Research Centre (CHRC), University of Évora, Évora, Portugal; ^7^Escola Superior de Enfermagem de São João de Deus, Universidade de Évora, Évora, Portugal; ^8^Grupo AFySE, Investigación en Actividad Física y Salud Escolar, Escuela de Pedagogía en Educación Física, Facultad de Educación, Universidad de Las Américas, Santiago, Chile

**Keywords:** physical literacy, older adults, physical activity, health literacy, community health

## Abstract

Population aging is a prominent phenomenon worldwide. The increase in physical inactivity and co-morbid diseases poses a major challenge to current community health policies. Physical activity guidelines recommended for older people have not been met by this population group. For this reason, a new model, physical literacy, is being innovated and has gained global attention and has emerged as an effective and innovative active aging strategy to improve physical activity participation of this vulnerable group. However, the evidence on physical literacy in the older adult so far is brief and diffuse. Therefore, the aim was to conduct a scoping review protocol to identify and map physical literacy in older people. This scoping review protocol was based on the Joanna Briggs Institute Method. The search will be performed on Embase, IBSS ProQuest, Medline OVID, PsycINFO Ebsco, PubMed, ScienceDirect, Scopus, SPORTDiscus, Social Services Abstracts ProQuest, Sociological Abstracts ProQuest, Web of Science ISI, Wiley Online Library, Cochrane Library, and ERIC Ebsco databases. All types of studies published since 2001 in English, Spanish, and Portuguese examining physical literacy over the lifespan of older adults were included. Two independent reviewers will organize and select studies according to the objectives and questions of the scoping review. The selected publications will be organized and summarized using a checklist proposed by the PRISMA-ScR. Qualitative data analysis (thematic analysis) will be performed to identify meanings and patterns to answer the research question. The final scoping review will present the main evidence available, key concepts/definitions, research conducted, and knowledge gaps related to physical literacy in older adults, leading to strategies to improve the community health of this population, as well as health literacy.

## 1 Introduction

Population aging is a real global challenge for government policies (1). There is concern about an aging population that is increasingly sedentary and whose co-morbid diseases increase health and community costs (1).

In developed countries, the population pyramid is inverted, with more and more older people and fewer children (2). So much so that in Spain, according to the projection of the National Institute of Statistics (2018–2068), in 2068 there could be more than 14 million people over 65 years of age, 29.4% of the total population (3).

Numerous studies have demonstrated the health benefits of physical activity (PA) for older people (4), reducing the prevalence of chronic conditions (5), improving mental health and cognition (6) as well as physical function (7), and reducing mortality rates (8).

However, the World Health Organization (WHO) PA guideline recommendations are failing because only 12% of the older person is adhering to them, with the older adult population being the most vulnerable (9).

In this sense, changes are taking place in the new attitudes toward physical inactivity in this population. Thus, physical literacy (PL) has emerged as one of the hot topics in education and public health for its power to promote an active lifestyle and to assess movement in relation to physical activity, quantifying motor skills, context, learning processes, and motivation (10).

PL was defined in the Bulletin of the International Council of Sport Science and Physical Education of the United Nations Educational, Scientific and Cultural Organization as the motivation, confidence, physical competence, knowledge and understanding to value and participate in a physically active lifestyle (11).

PL is an emerging concept that integrates different dimensions, which gives it a holistic view of physical development as a promoter of health (11). It is part of the lifelong learning process: The components interact integrally to facilitate a lifetime of participation and enjoyment of PA (12).

Several projects reflect the growing global awareness of the importance of PL for the health and wellbeing of communities (Sport for Life driven in Canada, in Sweden, IPLA from the United Kingdom and expanded to India, Australia, China, Japan, United States, rest of Europe, etc.). They also demonstrate the commitment of governments, organizations, and health and sport professionals to promote active and healthy lifestyles around the world. Each country tailor’s physical literacy initiatives according to its specific needs, resources, and cultural contexts (13). Studying the components of PL in older adults and understanding how they interact with each other could help facilitate lifelong participation and enjoyment of physical activities and therefore improve physical and mental health, prevent age-related injuries and diseases, improve quality of life, and increase independence (13).

Although PL plays an important role in promoting positive health habits (14), until today, little attention has been paid to its implications in this population (9). Older people could be more physically literate than younger generations (15), yet research studies on this special population are scarce. In other populations, physical activity practice has been associated with improved body composition (16), physical fitness, blood pressure, and health-related quality of life (HRQoL) (17).

There are recent reviews (13, 15) about PL in the older adult, but the concepts used to search for articles, the context, and the target population are different. Our study is exclusively for the population aged 65 and over, not as a recent review whose target population is adults and older adults including the population over 45 years old (13). There is also a new review of PL in older people, but its search concepts are quite broad (15); it incorporates physical activity, physical competence, and physical education, and our search has focused solely and exclusively on those that made direct reference to PL, following the guidelines of the review, which endorses multidimensional meaning of the original PL concept (18).

This review aimed to be a starting point to stimulate empirical research on PL in the older adult, as characterizing the development of its dimensions will enable the participation of this population in structured and full physical activities, adopting active lifestyles, promoting healthy aging, becoming a valuable tool for improving their quality of life, and maintaining good physical and mental health as they age, thus slowing down the sequelae inherent to the biological process, promoting their autonomy, and delaying the state of old age dependence.

Therefore, the aim was to carry out a scoping review protocol to identify and map PL in older people in different contexts (19).

## 2 Methods

### 2.1 Study design

This is a scoping review protocol study that will form the starting point for an exploratory project that systematically maps the available literature on the concept of PL in older adults. According to Munn et al. (19), scoping reviews use the scientific literature (1) to identify the types of available evidence in a given field, (2) to clarify key concepts/definitions in the literature, (3) to examine how research is conducted on a certain topic or field, (4) to identify key characteristics or factors related to a concept, (5) as a precursor to a systematic review, and (6) to identify and analyze knowledge gaps (20, 21). This protocol was developed using some of the items of the Preferred Reporting Items for Systematic Reviews and Meta-Analysis Protocols (PRISMA-P), and the future scoping review will be prepared adhering to the PRISMA extension for scoping reviews (PRISMA-ScR). This scoping review protocol was registered in the INPLASY (Code: INPLASY202330009) and performed according to the Joanna Briggs Institute (JBI) manual (20). Drafting of the protocol began in January 2023, and the scoping review is expected to be in May of the same year.

### 2.2 Review question

The PCC mnemonic is employed to shape a research question by considering the Population, Concept, and Context. This approach aids in pinpointing potential gaps in knowledge, understanding theories, highlighting crucial concepts, measuring specific aspects of interest, and elucidating the practices and evidence related to a particular topic (20). As a result, the review question will be formulated as such, “How is the concept of physical literacy characterized in older adults?”

P—People in their older adult years.
C—PL.
C—Any context.

It was decided to open the study question on the concept of PL in older adults in a context-independent manner to be able to reach all studies on this topic specifically in this population, which differs from previous studies (13, 15).

### 2.3 Eligibility criteria

Studies will be assessed for inclusion in the review according to the following criteria:

Study design: We will only include studies that investigate the PL throughout older adult life. This includes primary research (peer-reviewed research articles), evidence synthesis (narrative reviews, systematic reviews, scoping reviews, rapid reviews, etc.), conference abstracts, discussion articles, editorials, and thesis. We will not limit the included studies by the sample size of the study.
Outcomes: We will include studies examining outcomes under the concept of PL, both quantitatively and qualitatively.
Study population and additional characteristics: We will only include studies where the study population meets the MeSH (Medical Subject Headings) “Aged” characteristics: a person 65 years of age or older. We will not limit included studies by their ethnicity, country of origin, economic characteristics, or geographic region. We will limit the studies included by publication date to those published since 2001, since Whitehead’s PL concept was first described in that year (22). We will limit included studies to those published in English, Spanish, and Portuguese.

### 2.4 Information sources

We will search the information sources such as Embase, IBSS ProQuest, Medline OVID, PsycINFO Ebsco, PubMed, ScienceDirect, Scopus, SPORTDiscus, Social Services Abstracts ProQuest, Sociological Abstracts ProQuest, Web of Science ISI, Wiley Online Library, Cochrane Library, and ERIC Ebsco. Additional searches of gray literature will include the first 100 results of a Google Scholar search, hand searches, and contact with study authors. The reference list of relevant review papers and included articles were hand searched for additional articles. These searches will be carried out using the information source to which the Extremadura University and the Miguel Hernández University of Elche have access. We will persist in the search process until we have reached a high level of certainty that we have thoroughly considered all relevant studies associated with the review question. We will also search the registries in the International Platform of Registered Systematic Review and Meta-analysis Protocols (INPLASY) and the International Prospective Register of Systematic Reviews (PROSPERO) to identify planned, ongoing, or recently published reviews.

### 2.5 Search strategy

The specific literature search strategies will be developed after discussion and acceptance by the research team. As recommended in all types of JBI reviews, a three-step search strategy will be applied to reach the greatest number of publications and gray literature. Each step is specified in this section of the protocol.

#### 2.5.1 Identification of descriptors and keywords

The first step is an initial limited search of at least two appropriate online databases relevant to the topic. This initial literature search strategy will be developed using keywords and descriptors related to the topic, using in addition in the case of population a Medical Subject Headings (MeSH; Table 1).

**Table 1:** Descriptors used according to the Population, Concept, and Context.

Mnemonic	Keywords	MeSH
P (Population)	Aged, older adult, elderly, geriatrics and senior	Aged
C (Concept)	Physical literacy and physically literate	-
C (Context)	-	-

#### 2.5.2 Definition of data based

A second search should then be conducted using all identified keywords constituting the high-sensitivity search strategy across all sources of information included in Table 2. The search will also be carried out in Google Scholar (Gray literature). An age filter (Aged = 65+ years) will be applied to any source of information to optimize the search strategy. The search in the databases will be performed by two researchers in May 2023.

**Table 2:** Sources of information and high-sensitivity search strategies.

Source of information	Search strategy
Embase Aged (65+ years)	(‘physical literacy’ OR ‘physically literate’) AND (‘aged’ OR ‘older adults’ OR ‘elderly’ OR ‘geriatrics’ OR ‘seniors’)
International Bibliography of the Social Sciences	(“physical literacy” OR “physically literate”) AND (aged OR “older adults” OR elderly OR geriatrics OR seniors)
MEDLINE All aged (65 and over)”	((physical literacy* or physically literate*) and (aged or older adults* or elderly or geriatrics or seniors))
PsycINFO Aged (65 years and older)	(“physical literacy” OR “physically literate”) AND (aged OR “older adults” OR elderly OR geriatrics OR seniors)
PubMed Aged: 65+ years	(“physical literacy” OR “physically literate”) AND (aged OR “older adults” OR elderly OR geriatrics OR seniors)
ScienceDirect	(“physical literacy” OR “physically literate”) AND (aged OR “older adults” OR elderly OR geriatrics OR seniors)
Scopus	TITLE-ABS (“physical literacy” OR “physically literate”) AND (aged OR “older adults” OR elderly OR geriatrics OR seniors) AND (LIMIT-TO (LANGUAGE, “English”) OR LIMIT-TO (LANGUAGE, “Spanish”) OR LIMIT-TO (LANGUAGE, “Portuguese”))
SPORTDiscus	(“physical literacy” OR “physically literate”) AND (aged OR “older adults” OR elderly OR geriatrics OR seniors)
Social Services Abstracts	(“physical literacy” OR “physically literate”) AND (aged OR “older adults” OR elderly OR geriatrics OR seniors)
Sociological Abstracts	(“physical literacy” OR “physically literate”) AND (aged OR “older adults” OR elderly OR geriatrics OR seniors)
Web of Science ISI	(“physical literacy” OR “physically literate”) AND (aged OR “older adults” OR elderly OR geriatrics OR seniors)
Wiley Online Library	(“physical literacy” OR “physically literate”) AND (aged OR “older adults” OR elderly OR geriatrics OR seniors)
Cochrane Library	((“physical literacy” OR “physically literate”) AND (“aged” OR “older adults” OR “elderly” OR “geriatrics” OR “seniors”))
ERIC	(“physical literacy” OR “physically literate”) AND (aged OR “older adults” OR elderly OR geriatrics OR seniors)

#### 2.5.3 Search for additional sources in the references of publications

Third, the reference list of identified reports and articles should be searched for additional sources. In this third stage, the reference lists of all identified sources or only the reference lists of the sources that were selected from full text and/or included in the review can be examined. If necessary, the corresponding authors will be contacted by e-mail for further information.

### 2.6 Study selection

To avoid duplicate entries, the research results found through the search are entered into the EndNote software. In the first stage, two independent researchers will carry out the study selection by reviewing the title, abstract, and keywords. If there are uncertainties, they will refer to the full text, excluding studies that do not meet the predetermined eligibility criteria. If there are disagreements or doubts between the two researchers, a third researcher decides. The inclusion of any study might depend on using the critical appraisal tools provided by the JBI beforehand. The research outcomes and the study selection process will be documented in the scoping review, visually depicted through a Prisma Scoping Review® (23) flowchart. Separate appendices shall be included for details of sources included and a brief mention of sources excluded, and for excluded sources, the reasons for their exclusion shall be stated.

### 2.7 Data extraction and coding

Two separate reviewers (C.G.-A. and J.R.-C.) will independently screen articles, conduct the data extraction process (Data charting), and apply JBI critical appraisal tools. This approach aims to minimize errors and biases. This will provide the reader with a comprehensive and coherent summary detailing the specifics and characteristics of the studies, aligning closely with the objectives of the scoping review. An extraction tool in Microsoft Excel® will be employed, following the JBI methodology guidance for scoping reviews (as outlined in Table 3). The tool might undergo revisions during the process to further enhance comprehension of the subject matter (19).

**Table 3:** Data extraction tool to describe the details and characteristics of the studies.

Authors and year of publication	-
Country of origin	-
Population characteristics and sample size	-
Type of material	Peer-reviewed research articles, evidence synthesis, conference abstracts, discussion articles, editorials, and thesis.
Study design	Randomized or non-randomized controlled trials, cohort, case–control, cross-sectional, descriptive observational, ecological, or qualitative studies.
Theories or framework discussed	Health-promoting physical activity, motor competence, and phenomenological embodiment.
Aims/purpose	Description of the main objectives
Outcomes and details of these	Measurements related to the multifaceted concept comprised of affective (motivation and confidence), physical (physical competence), cognitive (knowledge and understanding), and behavioral (engagement in physical activities for life) domains
Results	-
Conclusions and key findings	-
Challenges and limitations	-

### 2.8 Analysis

Thematic analysis will be utilized for qualitative data analysis to uncover patterns and meanings that address the research question. Additionally, the study type and level of evidence from the study design will be assessed using the JBI Critical Appraisal Checklist.

### 2.9 Compiling, summarizing, and reporting the results

The final report will follow the PRISMA-ScR guidelines, utilizing tables, diagrams, and thematic maps to visually represent the synthesized evidence extracted from the data, facilitating a clearer understanding of the results. This phase will involve (1) analyzing the data, (2) presenting results connected to the research inquiries, and (3) interpreting the implications of these findings for further research and practical applications. As a result of interest, we expect to find information based on the conception, measurements, effectiveness, and/or testimonials related to older adults’ physical literacy/ies. A narrative summary will report the relationships between the results and the review objective and question and identify knowledge gaps for future studies (e.g., systematic reviews).

## 3 Study protocol timeline

The timeline for the study protocol of scooping review is going to develop in several phases, which are outlined below with the expected completion date for each phase.

1. Protocol Preparation

The organization of the protocol will be carried out during the month of January 2023.

1. Database search

The search of the aforementioned databases will be carried out during the months of February and March 2023.

1. Title and abstract screening

Two independent researchers will carry out the study selection by reviewing the title, abstract, and keywords in March 2023.

1. Full-text review

The revision of the full text is going to be carried out in April 2023.

1. Data extraction and analysis

Data extraction as well as data analyses will be performed by the authors in the month of April 2023.

1. Quality assessment

The quality assessment will be administered using the JBI Critical Appraisal Checklist during the month of April 2023.

1. Manuscript preparation and dissemination

Finally, the scooping review is expected to be prepared for dissemination and publication in May of the same year.

## 4 Discussion

The proposed scoping review aims to map and identify the available evidence regarding PL in older people in different contexts.

Delving into the concept of PL in Older Adults can make a difference in the development of active aging in the older population. It will have a positive impact on improving the health and HRQoL of this, ensuring healthy aging and providing the older adult with not only more years of life but also healthier years of life.

Therefore, this scooping review seeks to fill a scientific gap in PL in the older adult, as the reviews that exist so far on this concept, which has evolved as the understanding of physical education and the importance of physical activity have grown throughout the 20th century and continue into the 21st century (24) are not consistent on the target population we are addressing (13, 15). Moreover, it could be an effective active aging strategy to improve participation in PA of this population group in developed countries, consciously and motivated knowing the benefits of a healthy lifestyle, and guide the methodology of future lines of intervention for overall healthy aging.

## 5 Conclusion

This exploratory review aims to be a key point to enhance research on PL in older people, contributing to scientific production and guiding possible future studies to promote PL as a tool to improve active aging in the older population, enhancing community health literacy from PL. Regarding the findings, they will be articulated in a manuscript and published in a high-impact, open-access, peer-reviewed journal within the scientific community. Any modifications to the protocol will be justified and reported in the final scooping review publication.

## Author contributions

CG-A: Conceptualization, Investigation, Project administration, Writing – original draft preparation, Writing – review & editing. JR-R: Conceptualization, Data curation, Formal analysis, Investigation, Methodology, Project administration, Writing – original draft preparation, Writing – review & editing. JR-C: Investigation, Validation. JP: Visualization, Writing – review & editing. CF: Visualization, Writing – review & editing. AC-P: Funding acquisition, Supervision, Writing – review & editing. MS: Supervision, Visualization, Writing – review & editing. All authors contributed to the article and approved the submitted version.
